# Naturally Derived Malabaricone B as a Promising Bactericidal Candidate Targeting Multidrug-Resistant *Staphylococcus aureus* also Possess Synergistic Interactions with Clinical Antibiotics

**DOI:** 10.3390/antibiotics12101483

**Published:** 2023-09-26

**Authors:** Neethu Sivadas, Grace Kaul, Abdul Akhir, Manjulika Shukla, Murugan Govindakurup Govind, Mathew Dan, Kokkuvayil Vasu Radhakrishnan, Sidharth Chopra

**Affiliations:** 1 Chemical Sciences and Technology Division, CSIR-National Institute for Interdisciplinary Science and Technology, Thiruvananthapuram 695019, India; neethusiva90@gmail.com; 2 Academy of Scientific and Innovative Research (AcSIR), Ghaziabad 201002, India; grace.arv@gmail.com; 3 Division of Microbiology, CSIR-Central Drug Research Institute (CSIR-CDRI), Lucknow 226031, India; abd.bstdli@live.com (A.A.);; 4 Department of Plant Genetics Resource, Jawaharlal Nehru Tropical Botanic Garden and Research Institute, Palode, Thiruvananthapuram 695562, India

**Keywords:** malabariconeB, phenylacylphenols, multidrug resistance, *S. aureus*, synergy, natural products

## Abstract

The emergence of multidrug-resistant (MDR) superbugs underlines the urgent need for innovative treatment options to tackle resistant bacterial infections. The clinical efficacy of natural products directed our efforts towards developing new antibacterial leads from naturally abundant known chemical structures. The present study aimed to explore an unusual class of phenylacylphenols (malabaricones) from *Myristicamalabarica* as antibacterial agents. *In vitro* antibacterial activity was determined via broth microdilution, cell viability, time–kill kinetics, biofilm eradication, intracellular killing, and checkerboard assays. The efficacy was evaluated *in vivo* in murine neutropenic thigh and skin infection models. Confocal and SEM analyses were used for mechanistic studies. Among the tested isolates, malabaricone B (**NS-7**) demonstrated the best activity against *S. aureus* with a favorable selectivity index and concentration-dependent, rapid bactericidal killing kinetics. It displayed equal efficacy against MDR clinical isolates of *S. aureus* and *Enterococci*, efficiently clearing *S. aureus* in intracellular and biofilm tests, with no detectable resistance. In addition, **NS-7** synergized with daptomycin and gentamicin. *In vivo*, **NS-7** exhibited significant efficacy against *S. aureus* infection. Mechanistically, **NS-7** damaged *S. aureus* membrane integrity, resulting in the release of extracellular ATP. The results indicated that **NS-7** can act as a naturally derived bactericidal drug lead for anti-staphylococcal therapy.

## 1. Introduction

Multidrug-resistant (MDR) *Staphylococcus aureus* is now widely recognized as a severe burden to the healthcare system owing to its widespread occurrence and drug resistance profile. Nosocomial and community-acquired multidrug-resistant (MDR) *S. aureus* infections are a significant clinical concern globally, characterized by high morbidity and mortality rates. This is mostly attributed to their intrinsic resistance to commonly used antibiotics for treatment [1,2]. The development of various virulence factors and the rapid acquisition of the MDR phenotype over time make them outwit existing antibiotics, thereby creating life-threatening complications in infection control [3,4]. In light of the emergence of genetically variable epidemic strains of *S. aureus*, including methicillin-resistant *Staphylococcus aureus* (MRSA) and vancomycin-resistant *Staphylococcus aureus* (VRSA), and their increased incidence, the World Health Organization (WHO) declared them as “high priority” pathogens needing urgent attention [5]. This unceasing emergence and global spread of MDR pathogens necessitates the consistent discovery and development of new drug leads with novel target mechanisms to tackle MDR *S. aureus* infections.

Natural products and their analogs are pioneers as antibacterial agents and have been approved by the FDA for utilization in clinics [6].The characteristics of naturally derived compounds, such as unique chemo-diversity, bioavailability, target specificity, and microbial metabolism, have proved their ability to address the developing mechanisms of multidrug resistance in order to augment the drug discovery pipeline for antibiotics [7,8,9,10,11]. Therefore, the identification of novel chemical scaffolds from nature and their detailed characterization against predominant MDR strains is of great significance. In addition, their utilization in a synergistic combination with conventional antibiotics has emerged as a highly effective approach for infection control [12,13]. The highly enriched floristic diversity and unremitting legacy of utilizing naturally derived compounds for therapeutic advances [14,15,16] encouraged us to systematically explore the ability of known chemical scaffolds as promising candidates to fight MDR bacterial infections.

The present study investigated the antibacterial properties of a specific group of compounds belonging to the class of phenylacylphenols, derived from the *Myristicaceae* family, often known as nutmeg. Malabaricones, also known as phenylacylphenols or diarylnonanoids, are a distinctive category of secondary metabolites with bioactive properties. These compounds are synthesized by several plant species that are part of the well-known “*Myristicaceae*” family. The term “Malabaricones” was assigned to these compounds due to their initial discovery in the fruit rind of the *Myristica malabarica*, a wild nutmeg species [17]. Subsequently, it was determined that these bioactive elements are also present in other species of the *Myristica* and *Kenema* (limited)genera. *Myristicamalabarica* is a species that is recognized as red-listed and is endemic to specific regions. It is typically found in sub-tropical evergreen forests at altitudes of about 1000 m and in the southern and western ghats within *Myristica* swamps. Various components of this particular plant, such as the rind, mace, and seed, were discovered to have malabaricones as their primary phytoconstituents [18]. The primary components of malabaricones are acyl phenols of the 2,6-dihydroxyphenyl type and their derivatives. These compounds are characterized by a structure that includes a 2,6-dihydroxyacetophenone group connected to a benzene ring by a C_8_ alkyl chain [18].

In the present study, our attempts were directed towards developing the naturally derived chemical structure malabaricone B (**NS-7**) as a promising drug lead to target MDR-*S. aureus*. Herein, we demonstrated a comprehensive *in vitro* and *in vivo* evaluation of **NS-7** including the mechanistic studies. Also, for the first time, synergy studies of the compound with clinically used antibiotics were well established.

## 2. Results

### 2.1. Isolation of Malabaricones from the Fruit Rinds of M. malabarica Lam

**Figure 1:**
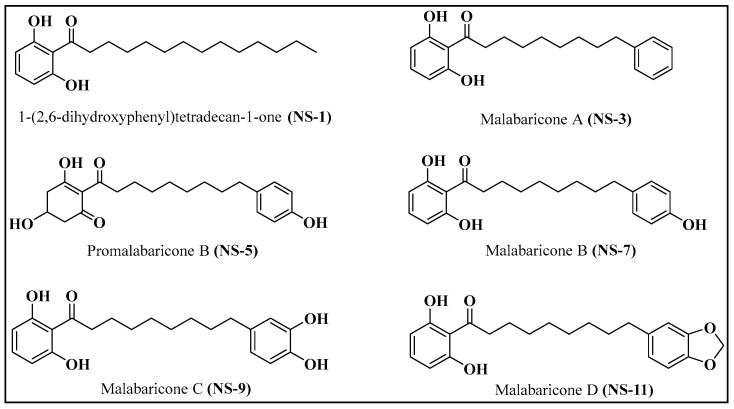
Structures of compounds isolated from *M. malabarica*.

The isolates from *M. malabarica* (Figure 1) were identified as the known chemical constituents, namely 1-(2,6-dihydroxyphenyl) tetradecan-1-one (**NS-1**), malabaricone A (**NS-3**), promalabaricone B (**NS-5**), malabaricone B (**NS-7**), malabaricone C (**NS-9**), and malabaricone D (**NS-11**), by interpreting their^1^H NMR, ^13^C NMR, and HRESI-MS spectroscopic data (available in the Supplementary Materials) and by comparing the results with relevant data from the literature.

### 2.2. Antibacterial Activity against Clinically Relevant Bacteria

To begin with, the antibacterial efficacy of the isolates was evaluated against ESKAPE pathogens. Amongst the isolates, **NS-7** displayed potent and specific activity with an MIC of 0.5 μg/mL, followed by **NS-11** and **NS-9**,against *S. aureus*. However, a significantly higher MIC (>128 μg/mL, Table 1) value of all tested compounds was a clear indication of their inactivity against Gram-negative bacteria (GNB). To confirm that the presence of an outer membrane (OM) is responsible for the lack of activity of **NS-7** against GNBs, its antibacterial activity was assessed with the addition of an OM permeabilizer, polymyxin B nonapeptide (PMBN), at non-lethal concentrations. As seen in Table 2, **NS-7** alone is inactive against GNBs with intact an OM but, in the presence of PMBN, its entry is facilitated due to increased membrane permeability, reflected in its lower MICs. The reduction in MIC of **NS-7** with the addition of PMBN against GNBs *A. baumannii* and *E. coli* (Table 2) clearly indicates that an intact OM is acting as a permeability barrier and that the target for **NS-7** is present across both bacterial species.

### 2.3. **NS-7** Imparted No Toxicity to Eukaryotic Cells

As we know, the cytotoxic effects of hit compounds have a key role in their further development as antibacterial agents. Intriguingly, the MTT assay results revealed that **NS-7** does not impart any toxicity to the host cells (Vero cells, ATCC CCL-81) and possessed an extremely favorable selectivity index (≥80, Table 3) which is highly promising for further characterization. On the other hand, **NS-9** and **NS-11** were cytotoxic and, thus, did not exhibit a favorable selectivity index, which precluded these compounds from further evaluation.

### 2.4. **NS-7** Is Active against MDR Clinical Strains of Staphylococci and Enterococci

With the selected, active, non-toxic compound **NS-7**, we further extended the study to explore its spectrum of activity against clinical MDR strains of *Staphylococci* and *Enterococci*. **NS-7** (MIC 1–2 μg/mL, Table 4) demonstrated equipotent activity against MRSA, VRSA, and vancomycin-resistant *Enterococcus* (VRE) strains. The admirable activity of **NS-7** against various MDR strains proved its ability to escape the prevalent drug resistant mechanisms, thereby acting as a promising candidate for treating MDR *S. aureus* and *Enterococcus* infections.

### 2.5. **NS-7** Possesses Rapid Bactericidal Activity

**Figure 2:**
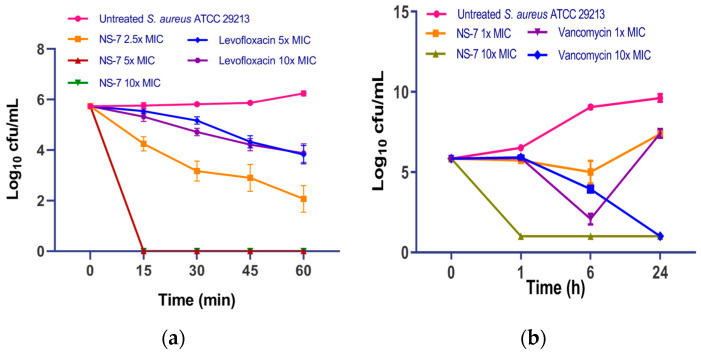
Bacterial time–kill kinetics of **NS-7** and comparators against *S. aureus* ATCC 29213 for (**a**) 1 h; (**b**) 24 h. The bacteria were incubated with various MICs for 24 h, then samples were removed at specified time intervals and the CFU was determined. The error bars indicate the standard deviations (SDs) derived from triplicate samples of each tested antibiotic or compound.

To determine the mode of action of **NS-7**, a bacterial time–kill kinetics assay with the compound was performed against *S. aureus*. Levofloxacin and vancomycin served as the reference standards. It was evidenced from the killing kinetics data (Figure 2) that **NS-7** demonstrates concentration-dependent, bactericidal activity against *S. aureus* ATCC 29213. At various MICs, **NS-7** showed rapid, bactericidal activity confirmed by complete eradication of viable cultures (~6 log_10_ cfu/mL, Figure 2a) within 15 min of incubation, and no regrowth in cultures could be seen up to 24 h (Figure 2b). At similar concentrations, levofloxacin caused only ~2 log_10_ cfu/mL reduction after 1 h of its exposure to *S. aureus*. The rapid killing kinetics of **NS-7** were also better than vancomycin at its 10X MIC (Figure 2b). The extremely fast bactericidal potency of **NS-7** with no regrowth was promising enough to carry forward for comprehensive evaluation.

### 2.6. Efficient Eradication of Preformed S. aureus Biofilm by **NS-7**

**Figure 3:**
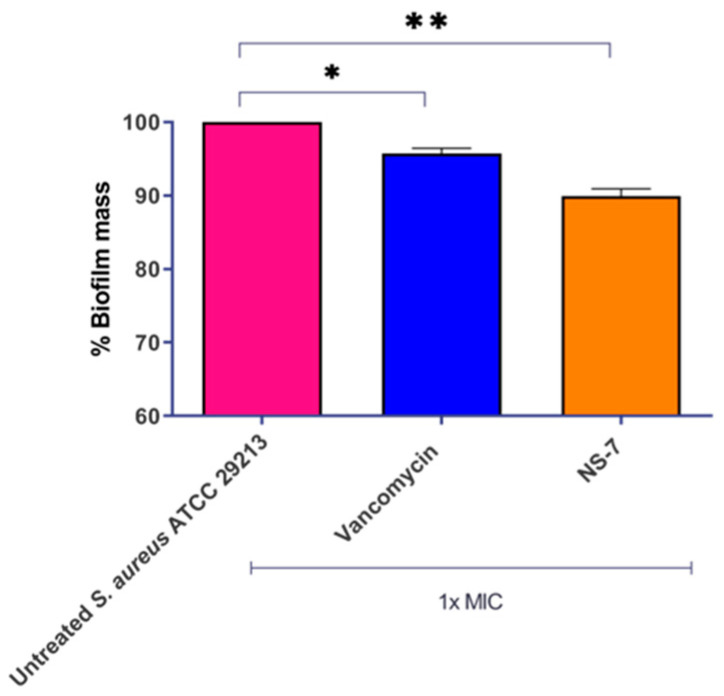
**NS-7** and vancomycin activity against *S. aureus* ATCC 29213 biofilm. Biofilm mass was stained with crystal violet post-treatment to quantify the % reduction, which is plotted. The error bars indicate the standard deviations (SDs) derived from triplicate samples of each tested antibiotic or compound. * (*p* < 0.5) and ** (*p* < 0.05) denotes significance.

Persistency and recurrence of *S. aureus* infections are due to its significant virulence mechanism through the formation of microbial biofilms. The presence of inherent tolerance in bacteria within the biofilm, coupled with the restricted diffusion of antibiotics across the surface, is the primary cause of biofilm-mediated AMR. This formidable resistance to conventional antibiotics and therapeutic failure demands an imperative need for new bactericidal agents capable of eradicating the biofilms [19,20]. In this regard, we evaluated the effect of **NS-7** in disrupting the *S. aureus* preformed biofilm. At 1X MIC, **NS-7** reduced ~10% of biofilm mass (Figure 3) which was better than vancomycin (~5%) at a similar concentration. Thus, **NS-7** exhibits rapid, bactericidal kinetics against planktonic *S. aureus* ATCC 29213 and is also effective against *S. aureus* in preformed biofilms.

### 2.7. **NS-7** Efficiently Cleared Intracellular S. aureus

**Figure 4:**
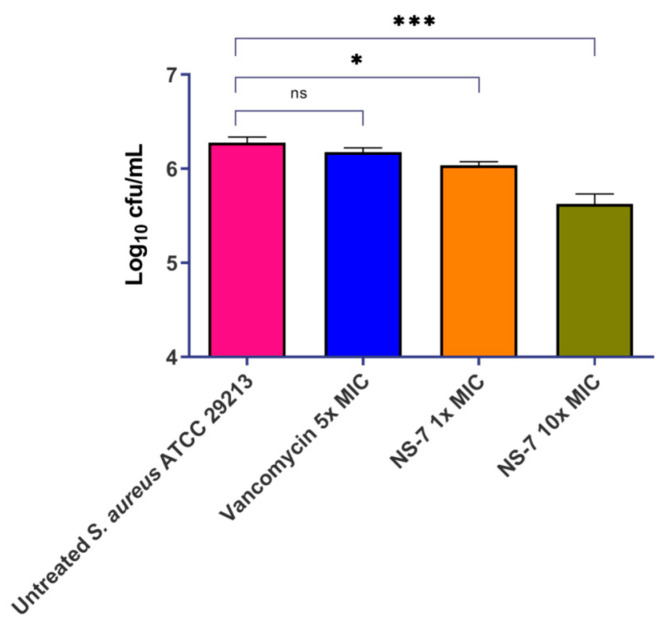
Activity of **NS-7** and comparators against intracellular *S. aureus* ATCC29213 in J774.A1 mouse macrophages. The error bars indicate the standard deviations (SDs) derived from triplicate samples of each tested antibiotic or compound. * (*p* < 0.5) and *** (*p* < 0.05) denotes significance.

Bacterial survival within phagocytic cells and inadequate cellular penetration of antibiotics creates serious complications in severe *S. aureus* infections. There is an urgent need for the development of effective antibacterial drugs that have significant penetrating power to specifically target and eliminate intracellular bacteria. This is crucial in order to avoid infections and combat the growing problem of antibiotic tolerance. Numerous natural and naturally derived compounds, synthetic compounds, and nanoparticles have demonstrated efficacy in the intracellular eradication of bacterial pathogens. [10]. In this context, we assessed the effect of **NS-7** against intracellular *S. aureus* in the murine macrophage cell line J774.A1.Vancomycin was used as the control antibiotic. **NS-7** exhibited bactericidal activity that was evidenced by a significant reduction in intracellular bacterial load at its 10X MIC treatment (~0.8 log_10_ cfu/mL reduction) as compared to untreated, which is better than vancomycin (~0.15 log_10_ cfu/mL reduction) at 5X MIC (Figure 4). Thus, **NS-7** is efficient in clearing intracellular bacteria much more potently than vancomycin. In addition, it also indicates that **NS-7** is able to achieve good cellular penetration and accumulation inside the macrophages, and that it is better than vancomycin.

### 2.8. **NS-7** Shows Synergism with Daptomycin and Gentamicin

**Figure 5:**
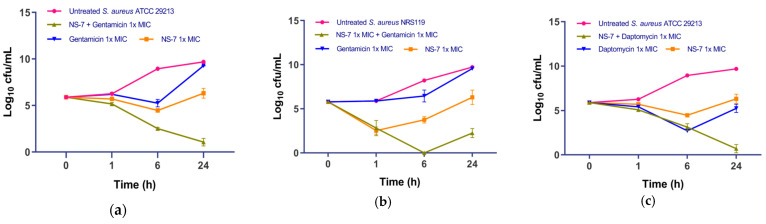
Bacterial killing kinetics of **NS-7** against *S. aureus* ATCC29213 with (**a**) gentamicin and (**b**) daptomycin; (**c**) bacterial killing kinetics of **NS-7** with gentamicin against gentamicin^R^ *S. aureus* NRS 119.The error bars indicate the standard deviations (SDs) derived from triplicate samples of each tested antibiotic or compound.

Recent advances in combination therapy to defeat MDR *S. aureus* infections emphasize the need to evaluate the synergistic interactions of a newly developed antibacterial agent with conventional antibiotics [21]. As such, the potential synergistic interactions between **NS-7** and clinically used antibiotics against *S. aureus* ATCC29213 were evaluated by determining the fractional inhibitory concentration (ΣFIC) using the checkerboard assay [22]. Among the tested antibiotics, **NS-7** showed synergistic interactions with gentamicin and daptomycin (FIC 0.265, Table 5). Furthermore, the bacterial killing kinetics of the active combinations were assessed in order to confirm the synergistic interactions. At 1X MIC against *S. aureus* ATCC 29213, **NS-7** + gentamicin caused a significant reduction in cfu/mL, better than either drug alone (Figure 5a), with the complete eradication of all culture in 24 h. When tested against gentamicin-resistant MRSA NRS 119 (Figure 5b), the combination of **NS-7** + gentamicin fared better than the individual drugs, with maximum activity achieved after 6 h of treatment (~8.2 log_10_ cfu/mL reduction as compared to untreated) with slight regrowth observed at 24 h. The combination of **NS-7** + daptomycin also performed better than either drug alone at 1X MIC and resulted in a ~9 log_10_ cfu/mL reduction in *S. aureus* ATCC 29213 (Figure 5c) in comparison to untreated control growth after 24 h. The results revealed that the antibacterial potential of **NS-7** can also be successfully employed with gentamicin and daptomycin to attain the augmented activity, even against gentamicin-resistant MRSA.

### 2.9. S. aureus Does Not Develop Resistance to **NS-7**

**Figure 6:**
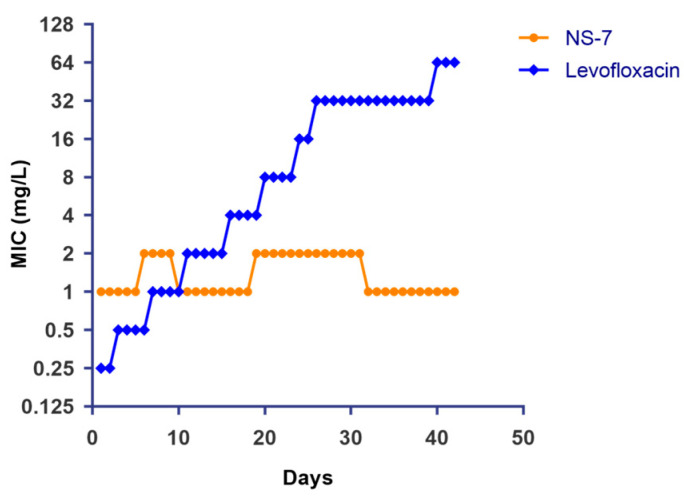
Induction of resistance in *S. aureus* ATCC29213 after continuous exposure to **NS-7** and levofloxacin at sub-MIC levels. The MIC of **NS-7** and levofloxacin against *S. aureus* during 40 serial passages was determined regularly and plotted.

In view of the escalating AMR globally, it is imperative to evaluate a newly developed antibacterial agent’s propensity for resistance development in bacteria. Therefore, the propensity of *S. aureus* to develop resistance to **NS-7** was evaluated via serial exposure (up to 40 days) to **NS-7** at sub-inhibitory concentrations along with levofloxacin as a control. Interestingly, the MIC of **NS-7** was not substantially altered after 40 passages, indicating that *S. aureus* lacks the ability to generate stable resistance to **NS-7** (Figure 6). In contrast, the MIC of the clinically used antibiotic levofloxacin increased 256-fold within the same time period, suggesting that **NS-7** can be an ideal antibacterial lead candidate and can act as an effective alternative to overcome drug resistance.

### 2.10. **NS-7** Displayed Prolonged PAE against S. aureus

A prolonged post-antibiotic effect (PAE) of an active antibacterial agent is an added advantage in reducing the dosage, which in turn guides the selection of antimicrobials for the desired clinical outcome. Herein, we assessed the persistent suppression of *S. aureus* growth (PAE) in the absence of **NS-7** after its 1 h treatment. Promisingly, **NS-7** exhibited an extended PAE of 1 h and >22 h when subjected to 1X and 10X MIC treatments, respectively, better than the control antibiotics vancomycin and levofloxacin (Table 6). Taken together, **NS-7** exhibits rapid bactericidal killing kinetics with potent activity against bacteria in various growth phases and a prolonged PAE.

### 2.11. **NS-7** Exerts Bactericidal Activity via Cell Membrane Lysis

**Figure 7:**
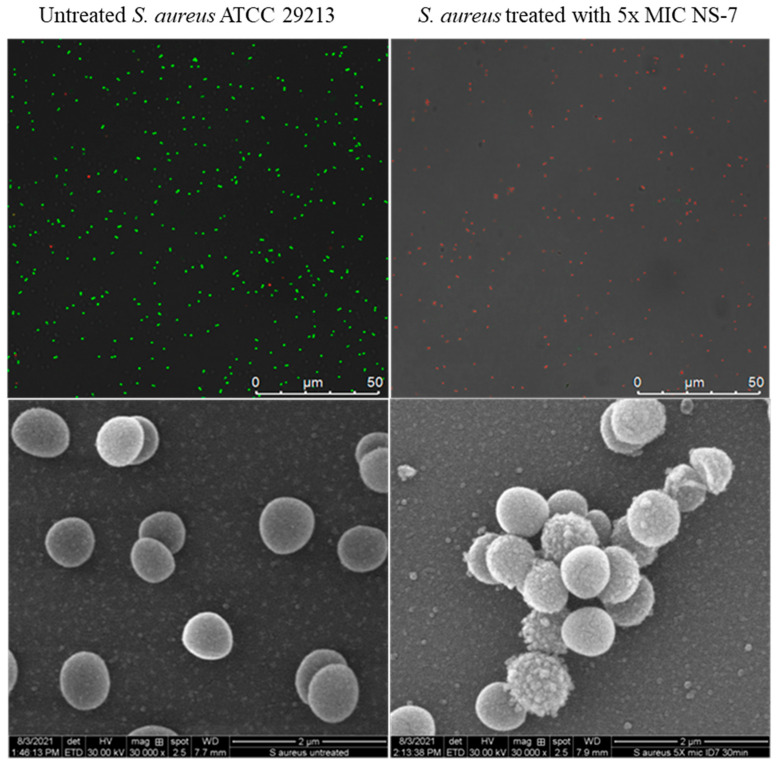
Confocal (**upper** panel) and SEM images (**lower** panel) of untreated and **NS-7**-treated *S. aureus* ATCC 29213at 5X MIC for 30 min. The cells were treated with **NS-7**, exposed to bacterial viability stains for confocal microscopy, and prepared for SEM imaging using the standard protocol. Viable bacterial cells retained SYTO9dye (green) whereas dead cells were stained red by PI, as seen under a confocal microscope. SEM images demonstrated membrane damage.

**Figure 8:**
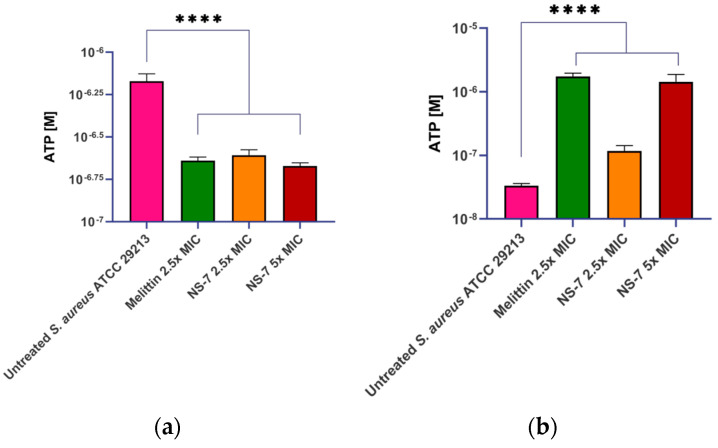
Treatment with **NS-7** leads to (**a**) intracellular ATP decrease; (**b)** extracellular ATP increase in **NS-7** treated *S. aureus* ATCC 29213. Melittin was used as the comparator. The error bars indicate the standard deviations (SDs) derived from triplicate samples of each tested antibiotic or compound. **** (*p* < 0.005) denotes significance.

To obtain an insight into the fast bactericidal activity of **NS-7** against *S. aureus*, confocal microscopic studies were performed using the LIVE/DEAD BacLight bacterial viability kit. It is evident from Figure 7 that dead cells (stained red) were predominant over viable cells upon treatment with 5X MIC of **NS-7** for 30 min, indicating a destructive effect on bacterial membrane integrity resulting in rapid bactericidal activity. Consistent with these results, scanning electron microscopy (SEM) images (Figure 7) exhibited the severely damaged morphology of *S. aureus* ATCC29213 exposed to **NS-7** (numerous lysed cells accompanied with cellular debris), with irregular cell wall structures and leakage of intracellular components in contrast to untreated *S. aureus* (intact morphology).To further ascertain the effect of **NS-7** on membrane integrity, the extra-and intracellular ATP levels in *S. aureus* upon treatment with **NS-7** were determined using an ATP bioluminescence assay kit. *S. aureus* ATCC 29213, when treated with 2.5X and 5X MIC of **NS-7** and with melittin as a control, demonstrated a significant dose-dependent decrease in intracellular ATP concomitant with an increase in extracellular ATP release(Figure 8a,b). The observed proportional increase and decrease in extracellular and intracellular ATP, respectively, implied that **NS-7** enhanced the permeability of the *S. aureus* cell membrane, resulting in the escape of ATP molecules. The results were comparable to treatment with melittin (a membrane-puncturing antimicrobial peptide), thus, indicating that the mode of action of the compound is by lysing membranes, leading to cell death. Taken together, the correlation between extracellular and intracellular ATP concentration along with the SEM data indicated that **NS-7** targeted the membrane integrity by disruption of the bacterial cell membrane, thus, triggering cell death.

### 2.12. **NS-7** Demonstrates Significant Efficacy in Murine Infection Models

**Figure 9:**
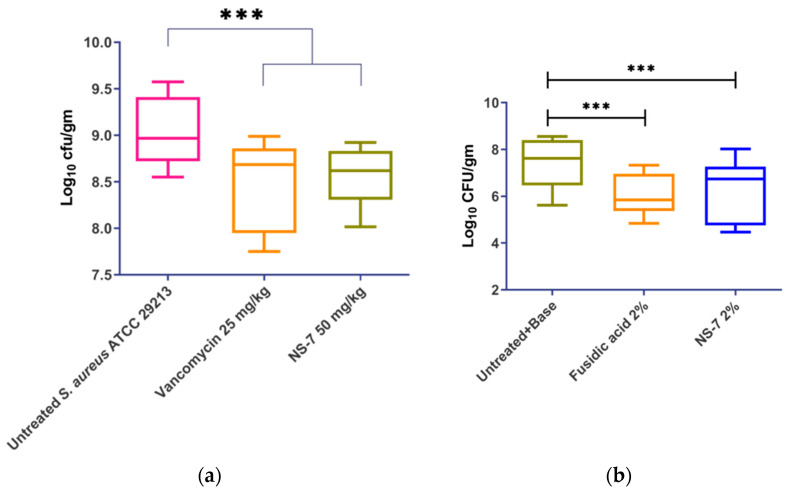
*In vivo* efficacy of **NS-7** and vancomycin in a murine model: (**a**) neutropenic thigh infection; (**b**) skin infection model. The error bars indicate the standard deviations (SDs) derived from triplicate samples of each antibiotic or compound treatment group tested in three independent experiments. *** (*p* < 0.05) denotes significance.

In view of **NS-7**’s potent activity against *S. aureus in vitro*, we tried to further establish its *in vivo* efficacy in murine infection models. The maximum tolerable dose (MTD) of **NS-7** was determined to be ≥250 mg/kg before testing in infection models. **NS-7** at 50 mg/kg demonstrated significant *in vivo* efficacy (Figure 9a) in a murine neutropenic thigh infection model as indicated by reduction of ~0.5 log_10_cfu/g bacterial load comparable to vancomycin at a b.i.d dose of 25 mg/kg.

In the skin infection model, the infected Swiss mice that received treatment twice daily with 2% **NS-7** (Figure 9b) exhibited a significant reduction in the bacterial burden (~1 log_10_cfu/g) of *S. aureus* ATCC29213 present in skin wounds. The compound exhibited comparable activity with the positive control of 2% fusidic acid that also caused a ~1.2 log_10_cfu/g reduction in bacterial load. The skin infection model revealed that **NS-7** can act as an excellent topical antibacterial agent to trigger wound healing in *S. aureus*-infected skin wounds, which is an added advantage. Altogether, **NS-7** proved its efficacy in both of the *in vivo* models, which in turn highlights its application as an effective antibiotic lead against severe infections caused by *S. aureus*. Additionally, **NS-7** is naturally derived and does not require any structural modifications, implying the possibility of a cost-effective drug development scheme to treat MDR *S. aureus* infections.

## 3. Discussion

The identification and development of new antibacterial agents with novel target mechanisms against the highly virulent MDR *S. aureus* strains are imperative to avoid the risk associated with resistant infections [23]. The unremitting legacy of utilizing naturally derived compounds for therapeutic advance emphasized exploring the ability of known chemical scaffolds as promising candidates to combat MDR *S. aureus* infections [14].

Malabaricones have gained adequate attention owing to their structural characteristics and broad array of pharmacological activities, including antioxidant [24], anti-cancer [25,26,27,28], anti-inflammatory [29,30], anti-hypertensive [31], anti-fungal [32], etc. The first report on the antibacterial activity of malabaricones (B and C) isolated from the mace of *Myristica fragrans* against microorganisms (*S. aureus*, *B. subtilis*, *S. durans*, and *C. albicans*) appeared many years ago [33], indicating the potent activity of malabaricone B against *S. aureus* compared to malabaricone C. It also revealed some structure–activity relationships responsible for antibacterial activity. Surprisingly, apart from this preliminary screening, malabaricone B has not been extensively studied to establish its antibacterial properties since then, while few studies have been published regarding the antimicrobial actions of malabaricone C, including Arg-gingipain inhibition [34], anti-quorum sensing activity [35], and larvicidal activity [36].

In this perspective, the present study focused on the detailed biological characterization of the rare phenylacylphenol class compound malabaricone B (**NS-7**) from the *Myristicaceae* (the nutmeg) family. To begin with, naturally known abundant malabaricones were isolated from the fruit rind of *M. malabarica,* which is identified as the reservoir of these secondary metabolites. The hit compound **NS-7**, with a favorable SI, exhibited excellent activity against multiple MDR strains of *S. aureus* and *Enterococcus*, demonstrating its ability to circumvent existing resistance mechanisms and its lack of cross-resistance. In the presence of PMBN, an OM permeabilizer, **NS-7** could act against the GNBs *E. coli* and MDR *A. baumannii*, indicating its broad-spectrum activity and the probable presence of a common target. Additionally, as **NS-7** contains a phenolic part, it might be reduced by the thioredoxin system, which makes Gram-positive bacteria more susceptible to it as compared to Gram-negative bacterial pathogens. **NS-7** exhibited rapid bactericidal killing kinetics that were reflected in its low propensity for resistance induction in *S. aureus*.

**NS-7** also caused efficient disruption of established *S. aureus* biofilm at low concentrations, along with good cellular penetration in acting against intracellular *S. aureus*. The ability of **NS-7** to act against *S. aureus*’s indifferent metabolic states makes it a good candidate for further development as an antibacterial lead. Its prolonged PAE, which can be attributed to its rapid bactericidal activity, is an added advantage for further developing it as a potent antibacterial drug. A longer PAE favors a short exposure time to attain superior activity with a lower dosage administration [37]. Mechanistic studies via confocal microscopy and SEM indicated membrane damage at bactericidal concentrations of **NS-7**, corroborated by the extracellular release of large macromolecules, such as ATP, ultimately leading to bacterial cell death.

Recent advances in combination therapy to defeat life-threatening diseases indicate the importance of synergism between drugs for pronounced effect. **NS-7** promisingly synergized with gentamicin and daptomycin, outcompeting the drug-alone groups against both sensitive and gentamicin-resistant *S. aureus*. Its impressive *in vitro* antibacterial profile prompted us to determine its *in vivo* efficacy in murine neutropenic thigh and skin infection models. With a maximum tolerable dose of ≥250 mg/kg in mice, **NS-7** exhibited good efficacy in both the infection models at much lower doses, indicating its potential as an antibacterial lead candidate.

Thus, we have developed malabaricone B as a new antibacterial drug candidate targeting MDR and persistent *S. aureus* infections.

## 4. Materials and Methods

### 4.1. Plant Material

*Myristica malabarica* fruits were collected from the Chemunji Hills of the Agasthyamalai Biosphere Reserve in Thiruvananthapuram District, Kerala, India. Dr. Mathew Dan (plant taxonomist, JNTBGRI, Palode, Trivandrum, India) authenticated the collected material, and a voucher specimen [*Myristicamalabarica*, Chemunji Hills, Trivandrum, Kerala, April, 2017, Govind, 83442 (TBGT)] was deposited in the institute’s herbarium.

### 4.2. Extraction and Isolation

Initially, rinds were separated from the fruits and air-dried. The grounded material (670 g) was extracted with dichloromethane (2 L × 3 days). The crude extract was obtained by decanting, filtering, and evaporating the supernatant under reduced pressure in a Heidolph rotary evaporator. Further fractionation of the crude extract (25 g) over 100–200 mesh silica gel by gradient elution with hexane/ethylacetate (100:0 to 0:100, *v*/*v*) yielded 60 fractions. Based on the similarities in TLC, these fractions were combined together, and repeated column chromatic separation followed by crystallization techniques resulted in the isolation of pure compounds.

### 4.3. Reagents and Growth Media

Bacterial culture media (MHB, MHBII, and TSB) were purchased from Becton-Dickinson (Franklin Lakes, NJ, USA). All antibiotics and chemicals used in the studies were obtained from Sigma-Aldrich (St. Louis, MO, USA). Eukaryotic cell culture growth media RPMI and FBS were purchased from Gibco (Waltham, MA, USA).

### 4.4. Bacterial Strains

The ESKAPE panel comprised Escherichia coli ATCC 25922, *Staphylococcus aureus* ATCC 29213, Klebsiella pneumoniae BAA-1705, Acinetobacter baumannii BAA-1605, Pseudomonas aeruginosa ATCC 27853, and Enterococcus NR 31903. The clinical MDR *S. aureus* and Enterococcus strain details are provided in Table 4. All strains were procured from the American Type Culture Collection (ATCC, Manassas, VA, USA) and Biodefense and Emerging Infectious Disease/Network on Antimicrobial Resistance in *Staphylococcus aureus* (BEI/NARSA), and were routinely cultivated on appropriate culture media, namely MHB II, MHA, TSB, or TSA.

### 4.5. Antibacterial Susceptibility Testing against ESKAPE Panel of Bacteria

The antibacterial activity of test compounds was assessed against an ESKAPE pathogen panel via broth microdilution assay using standard CLSI guidelines [38], as described before. The MIC of each test compound was determined through three replicate measurements.

### 4.6. Outer Membrane Susceptibility Assay

As previously described, polymyxin B nonapeptide (PMBN) was used at 10 µg/mL in the culture medium to determine the susceptibility of Gram-negative bacteria-*E. coli* and *A. baumannii* towards the tested compounds via broth microdilution assay [39]. Levofloxacin, vancomycin, and rifampicin served as controls.

### 4.7. Cell Viability Assay in Vero Cells

To test the effect of compounds on the growth of mammalian cells, a cell viability assay was performed using MTT, as per published protocols [40]. The SI (selectivity index) was calculated as CC_50_/MIC, where CC_50_ is defined as the compound concentration resulting in 50% reduction in cell viability post-treatment. The experiments were performed thrice, and the average mean was used to calculate CC_50_ and SI.

### 4.8. Activity against MDR Strains of S. aureus and Enterococcus Panel

**NS-7** was tested against clinical MDR *S. aureus* and *Enterococcus* strains comprising of MSSA, MRSA, VRSA, VSE, and VRE via broth microdilution assay. The resistance profile of each strain is described in Table 4.

### 4.9. Time–Kill Kinetics Study

The killing kinetics of compound were determined via time–kill kinetics assay according to CLSI guidelines, following a previously described protocol [40]. Treated and untreated *S. aureus* samples were withdrawn at 0 min, 15 min, 30 min, 45 min, 60 min, 6 h and 24 h, and the cfu/mL at each time point was determined and plotted with respect to time. Experiments were performed in triplicate and the mean data with SD were plotted.

### 4.10. Biofilm Eradication Assay

*In vitro S. aureus* biofilms were grown and treated with **NS-7** and vancomycin at 1X MIC using the method described before [41]. A total of 0.06% crystal violet was used to stain the remaining biofilm mass post-treatment, while 30% acetic acid (0.2 mL/well) was utilized to perform the elution, and quantification was performed by measuring the absorbance at 600 nm. Experiments were repeated thrice, and the average mean was plotted with SD.

### 4.11. Intracellular Activity Assay

The murine macrophage cell line J774.A1 was used to establish intracellular *S. aureus* infection, and the ability of **NS-7** to kill *S. aureus* inside host eukaryotic cells was determined according to the previously described protocol [42]. Macrophages were treated with 1X and 10X MIC of **NS-7**, while vancomycin at 5X MIC and untreated macrophages served as controls. Experiments were repeated thrice, and the average mean along with the SD were plotted for each group.

### 4.12. Synergy Screening of **NS-7** with Frontline Antibiotics

A checkerboard assay was used to determine fractional inhibitory concentrations (ΣFICs) as a measure of synergy between the test compound and clinical antibiotics in combination [40]. The antibiotics that were examined in this study encompassed daptomycin, gentamicin, levofloxacin, linezolid, meropenem, minocycline, rifampicin, and vancomycin. The summation of fractional inhibitory concentrations (ΣFIC) was determined using the following formula: ΣFIC = FIC A + FIC B. Here, FIC A represents the ratio of the MIC of compound “A” in combination with antibiotic “B” to the MIC of compound “A” alone, while FIC B represents the ratio of the MIC of antibiotic “B” in combination with compound “A” to the MIC of antibiotic “B” alone. The combination is deemed synergistic when the ΣFIC is equal to or less than 0.5. It is considered indifferent when the ΣFIC is greater than 0.5 but less than or equal to 4. Lastly, it is classified as antagonistic when the ΣFIC exceeds 4 [22].

### 4.13. Induced Resistant Mutant Generation Studies

*S. aureus* ATCC29213 was subjected to 40 days of serial passaging in the presence of subinhibitory concentrations of **NS-7** and levofloxacin to induce resistance. The changes in MIC were monitored every third passage and plotted against the number of passages, and the fold increase in MIC was calculated as previously described [43].

### 4.14. Determination of Post-Antibiotic Effect (PAE)

*S. aureus* ATCC29213 grown overnight and subsequently diluted to ~10^5^ cfu/mL was subjected to 1X and 5X MIC of **NS-7** and the control antibiotics vancomycin and levofloxacin for 1 h. PAE was assessed in accordance with the previously outlined protocol [37,41].

### 4.15. Bacterial LIVE/DEAD BacLight Assay

The LIVE/DEAD BacLight bacterial viability kit (Invitrogen, Waltham, MA, USA, catalogue no. L7007) was used to assess the viability of *S. aureus* ATCC29213 upon treatment with **NS-7**, as described in the manufacturer guidelines. The method consists of two fluorescent probes SYT09 (the green intercalating stain permeates the membranes of both live and dead cells) and propidium iodide (PI; the red intercalating stain enters only the cells with damaged membranes) with excitation/emission wavelengths corresponding to 488/530 nm and 488/620 nm, respectively. Initially, *S. aureus* cells were adjusted to OD_600_ = 0.2 in PBS buffer and were exposed to **NS-7** at 5X MIC for 10−30 min. After the compound exposure the bacterial suspensions were washed in PBS buffer and incubated with dye mixture of SYTO9 and PI (mixing equal volume of each) for 20 min in the dark at 37 °C. An aliquot of the stained sample was taken, and the fluorescent images were examined by using a Leica SP8 laser scanning confocal microscope (Leica, Wetzlar, Germany).

### 4.16. Scanning Electron Microscopy (SEM)

Scanning electron microscopy was used to examine the surface morphological changes in *S. aureus* ATCC29213 cells. Briefly, the mid-logarithmic-phase *S. aureus* cells (~10^8^ cfu/mL) were treated with 5X MIC of **NS-7** at 37 °C for 30 min with untreated bacterial cells as controls. After the incubation period, the bacterial cells were washed two times by using PBS (pH 7.4) and fixed overnight with 2.5% glutaraldehyde in PBS at 4 °C. The next day, the cells were dehydrated in a series of graded ethanol (20–95%) and finally dissolved in pure ethanol and vacuum-dried. The dried cells were gold-coated with an automatic sputter coater and, finally, the images of the samples were recorded by using Quanta 3D 250 field-emission scanning electron microscopy at 30 kV for *S. aureus.*

### 4.17. Determination of Extracellular and Intracellular ATP

An ATP Bioluminescence Assay Kit CLS II (Roche, Basel, Switzerland, Cat. No. 11699695001) was used to measure the extracellular and intracellular ATP levels of *S. aureus* in accordance with the manufacturer’s instructions and the previously described protocol [40]. Briefly, *S. aureus* ATCC29213 culture in log phase was treated with 2.5X and 5X MIC of **NS-7** and 2.5X melittin to determine extracellular and intracellular ATP levels. The luciferase agent was added to the extracellular and intracellular samples in equal volumes. This addition was carried out using automated injection into 96-well white plates. The signals obtained were then integrated for a duration of 1 to 10 s using the Promega GloMax^®®^ 96 Microplate Luminometer.

### 4.18. Murine Neutropenic Thigh Infection Model

Here, 4–5 weeks old BALB/c mice weighing~22–24 g were grouped together in a set of 5/per cage. Mice were rendered neutropenic, infected with (10^7^–10^9^ cfu/mL) *S. aureus* intramuscularly, treated, and sacrificed according to the previously described protocol [44]. Experimental groups were **NS-7** (50 mg/kg), vancomycin (25 mg/Kg),and untreated controls that were administered saline. Post-sacrifice, each removed thigh was homogenized in 5 mL of PBS, serial dilutions were performed, and the homogenized samples were plated on Mueller–Hinton agar plates to quantify the colony-forming units (cfu). Following incubation at a temperature of 37 °C for a duration of 18–24 h, the cfu were quantified. The experiments were conducted in triplicate, and the resulting mean data were graphed along with standard deviation (SD) from all three experiments.

### 4.19. Murine Skin Infection Model

This *in vivo* study utilized 6-week-old male BALB/c mice in accordance with the previously established protocol [40]. The experimental groups consisted of untreated control mice, mice treated with 2% fusidic acid (serving as the positive control), mice treated with 2% **NS-7** (the test drug), and mice treated with the vehicle (base). The experiments were conducted three times, and the mean log_10_cfu/g was determined for each experiment. The average mean and SD were then graphed based on the results obtained from all three experiments.

## 5. Conclusions

To conclude, the present study illustrates the development of a known natural product, malabaricone B (**NS-7**), a phenylacylphenol from the fruit rinds of *Myristic malabarica*, as a membrane-active, rapid, bactericidal agent equipotently active against MDR *S. aureus* without any inducible resistance. S-7 demonstrates significant activity against clinical MDR strains of *S. aureus* and *Enterococcus,* lacks cytotoxicity towards eukaryotic cells, efficiently eradicates preformed biofilms, and is highly efficient in killing intracellular *S. aureus*. In addition, **NS-7** exhibits excellent synergistic interactions with daptomycin and gentamicin against *S. aureus* ATCC 29213, including gentamicin-resistant MRSA NRS119, and demonstrates its *in vivo* potential by reducing the bacterial load in both *S. aureus*-infected neutropenic thigh infection and skin infection models. The rapid bactericidal potential of **NS-7** proceeds via lysing the bacterial cell membrane suggesting it to be a membrane-active antibacterial agent. Taken together, **NS-7** exhibits all the desired properties to be efficiently translated as a potent anti-staphylococcal therapeutic.

## 6. Patents

The work reported here has been duly filed for an Indian patent with application number 202111044825.

## Figures and Tables

**Table 1:** MIC (µg/mL) table for *M. malabarica* isolates against ESKAPE pathogen panel.

Bacterial Strains	NS-1	NS-3	NS-5	NS-7	NS-9	NS-11	Levofloxacin
*S. aureus* ATCC 29213	>128	>128	16	0.5	4	1	0.125
*Enterococcus* NR 31903	NT	NT	NT	2	NT	NT	>64
*K. pneumoniae* BAA 1705	>128	>128	>128	>128	>128	>128	64
*A. baumannii* BAA 1605	>128	>128	>128	>128	>128	>128	8
*P. aeruginosa* ATCC 27853	>128	>128	>128	>128	>128	>128	0.5
*E. coli* ATCC 25922	>128	>128	>128	>128	>128	>128	0.03

NT: Not tested.

**Table 2:** MIC (µg/mL) table for **NS-7** with PMBN against Gram-negative pathogens.

Bacterial Strains	MIC (µg/mL)								
	PMBN	NS-7		Rifampicin		Vancomycin		Levofloxacin	
		PMBN		PMBN		PMBN		PMBN	
		(−)	(+)	(−)	(+)	(−)	(+)	(−)	(+)
*E. coli* (ATCC 25922)	>64	>512	2	8	0.06	512	128	0.03	0.015
*A. baumannii* (ATCC BAA-1605)	>64	>512	4	2	0.03	256	64	8	8

**Table 3:** Selectivity index of **NS-7** against Vero cells, ATCC CCL-81.

Compound	MIC (µg/mL) against *S. aureus* ATCC 29213	CC_50_ (µg/mL)	Selectivity Index (CC_50_/MIC)
**NS-7**	0.5	>40	>80

**Table 4:** MIC (µg/mL) table for **NS-7** and comparators against a panel of *S. aureus* and *Enterococcus* MDR clinical strains.

Strains		MIC (µg/mL)			
		NS-7	Levofloxacin	Methicillin	Vancomycin
**MSSA**	*S. aureus* ATCC 29213	1	0.125	1	1
**MRSA**	NRS 100	1	0.125	>64	2
	NRS 119	1	16	>64	2
	NRS 129	1	0.125	64	1
	NRS198	1	32	>64	2
	NRS192	1	8	>64	1
	NRS191	1	32	>64	2
	NRS193	2	32	>64	2
	NRS186	2	8	64	2
	NRS194	1	0.125	16	1
**VRSA**	VRS 1	1	64	>64	>64
	VRS 4	2	>64	>64	>64
	VRS 12	2	64	64	>64
**VSE**	NR 31884	2	0.5	64	2
	NR 31885	2	0.5	>64	2
	NR 31886	2	0.5	>64	2
	NR 31887	2	0.5	16	1
	NR 31888	2	0.5	>64	1
**VRE**	NR 31903	2	>64	>64	>64
	NR 31909	2	>64	>64	>64
	NR 31912	1	>64	>64	>64

MSSA: methicillin-susceptible *Staphylococcus aureus*; MRSA: methicillin-resistant *Staphylococcus aureus*; VRSA: vancomycin-resistant *Staphylococcus aureus*; VSE: vancomycin-susceptible Enterococcus faecalis; VRE: vancomycin-resistant Enterococcus faecium.

**Table 5:** Synergy studies of **NS-7** with FDA-approved antibiotics against *S. aureus* ATCC 29213.

Compound/Drug	MIC (µg/mL)	MIC (µg/mL) of NS-7 “A” in Combination with Antibiotic	MIC (µg/mL) of Antibiotic “B” in Combination with NS-7	FIC A	FIC B	ΣFIC (FIC A + FIC B)	Inference
**NS-7**	2						
Gentamicin	0.5	0.06	0.125	0.03	0.25	0.28	Synergy
Daptomycin	1	0.06	0.25	0.03	0.25	0.28	Synergy
Ceftazidime	16	2	16	1	1	2	No interaction
Levofloxacin	0.25	2	0.25	1	1	2	No interaction
Linezolid	4	1	2	0.5	0.5	1	No interaction
Meropenem	1	2	1	1	1	2	No interaction
Minocycline	0.25	2	0.25	1	1	2	No interaction
Rifampicin	0.0078	2	0.0078	1	1	2	No interaction
Vancomycin	1	1	0.5	0.5	0.5	1	No interaction

**Table 6:** Post-antibiotic effect (PAE) of **NS-7** against *S*. *aureus* ATCC 29213.

	Untreated	Levofloxacin 1X	Levofloxacin 10X	Vancomycin 1X	Vancomycin 10X	NS-7 1X	NS-7 10X
Time for 1 log_10_ (h)	~2	~2.5	~3.5	~3	~3.5	~3	>24
PAE (h)	0	~0.5	~1.5	~1	~1.5	~1	>22

## Data Availability

The data presented in this study are available in the manuscript and supplementary material.

## Supplementary Materials

The following supporting information can be downloaded at: https://www.mdpi.com/article/10.3390/antibiotics12101483/s1, Figure S1: Schematic representation of extraction and isolation procedure from rind; Figures S2, S4, S6, S8, S10, and S12: ^1^H NMR spectrum of **NS-1**, **NS-3**, **NS-5**, **NS-7**, **NS-9**, and **NS-11**, respectively; Figures S3, S5, S7, S9, S11, and S13: ^13^C NMR spectrum of **NS-1**, **NS-3**, **NS-5**, **NS-7**, **NS-9**, and **NS-11**, respectively.
