# Supplementary material for: Naturally Derived Malabaricone B as a Promising Bactericidal Candidate Targeting Multidrug-Resistant Staphylococcus aureus also Possess Synergistic Interactions with Clinical Antibiotics
Source: Antibiotics (Basel). 2023 Sep 26;12(10):1483. doi: 10.3390/antibiotics12101483 (PMC10604362; doi:10.3390/antibiotics12101483)
Supplement: Supplementary file 1 [file antibiotics-12-01483-s001.zip › antibiotics-2597422-supplementary.pdf]

# Supplementary Materials

# Naturally Derived Malabaricone B as a Promising Bactericidal Candidate Targeting Multidrug-Resistant *Staphylococcus aureus*, also possess Synergistic Interactions with Clinical Antibiotics

**Neethu Sivadas [1,2,‡], Grace Kaul [2,3,‡], Abdul Akhir [3], Manjulika Shukla [3], Murugan Govindakurup Govind [4], Mathew Dan [4], Kokkuvayil Vasu Radhakrishnan [1,2,*] and Sidharth Chopra [2,3,*]**

[1] Chemical Sciences and Technology Division, CSIR-National Institute for Interdisciplinary Science and Technology, Thiruvananthapuram-695019, India
[2] Academy of Scientific and Innovative Research (AcSIR) Ghaziabad-201002, India
[3] Division of Microbiology, CSIR-Central Drug Research Institute (CSIR-CDRI), Lucknow - 226031, Uttar Pradesh, India
[4] Department of Plant Genetics Resource, Jawaharlal Nehru Tropical Botanic Garden and Research Institute, Palode, Thiruvananthapuram-695562, India
‡ These two authors contributed equally to this work.
* Correspondence:
Dr. Sidharth Chopra, Associate Professor, Division of Microbiology, CSIR-Central Drug Research Institute, Sector 10, Janakipuram Extension, Sitapur Road, Lucknow - 226031, Uttar Pradesh, India. Tel: +91-522-2772450, Fax: +91-522-2771941
Email: skchopra007@gmail.com.
Dr. Kokkuvayil Vasu Radhakrishnan, Senior Principal Scientist, Chemical Sciences and Technology Division, CSIR-National Institute for Interdisciplinary Science and Technology, Thiruvananthapuram-695019, Kerala, India. Tel: +91-471-2515226, Fax: +91-471-2491712.
Email: radhu2005@gmail.com.

**CONTENTS**


**Figure S1:** Schematic representation of extraction and isolation procedure from rind **3**

**Figure S2:** $^{1}$H NMR spectrum of NS-1 **4**

**Figure S3:** $^{13}$C NMR spectrum of NS-1 **4**

**Figure S4:** $^{1}$H NMR spectrum of NS-3 **5**

**Figure S5:** $^{13}$C NMR spectrum of NS-3 **6**

**Figure S6:** $^{1}$H NMR spectrum of NS-5 **7**

**Figure S7:** $^{13}$C NMR spectrum of NS-5 **7**

**Figure S8:** $^{1}$H NMR spectrum of NS-7 **8**

**Figure S9:** $^{13}$C NMR spectrum of NS-7 **9**

**Figure S10:** $^{1}$H NMR spectrum of NS-9 **10**

**Figure S11:** $^{13}$C NMR spectrum of NS-9 **10**

**Figure S12:** $^{1}$H NMR spectrum of NS-11 **11**

**Figure S13:** $^{13}$C NMR spectrum of NS-11 **12**

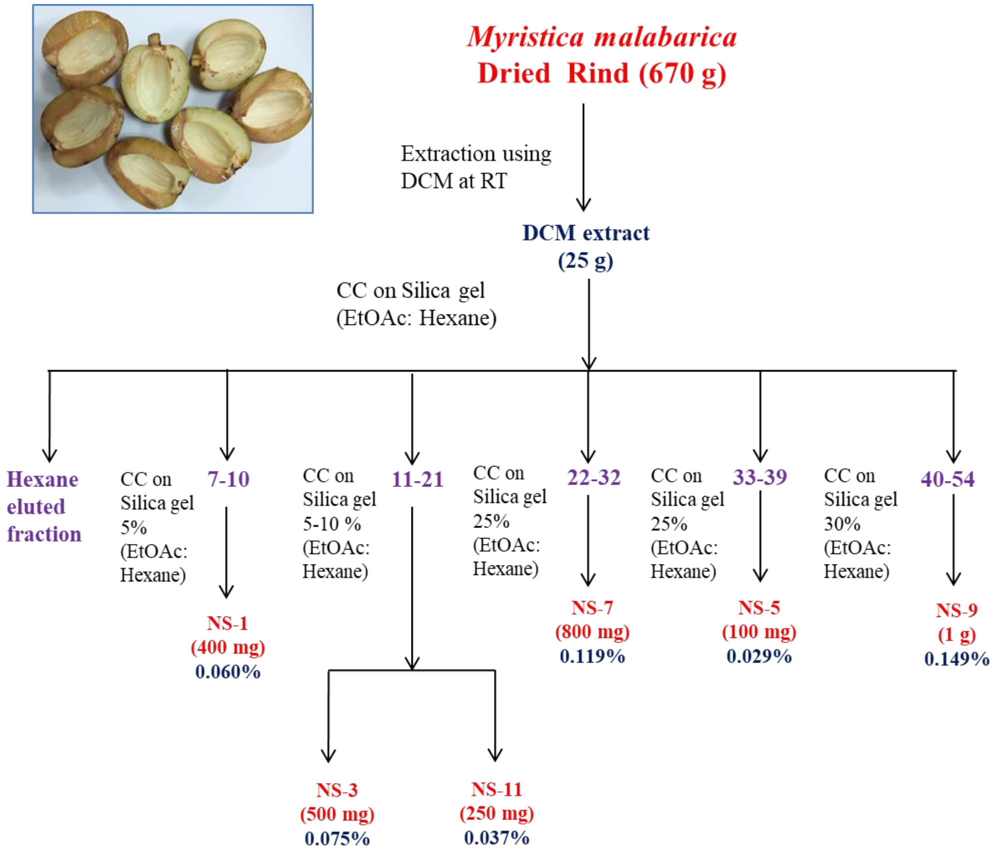


**Figure S1.** Schematic representation of extraction and isolation procedure from the rind

**Spectral Data**

1-(2, 6-dihydroxyphenyl) tetradecan-1-one (**NS-1**)

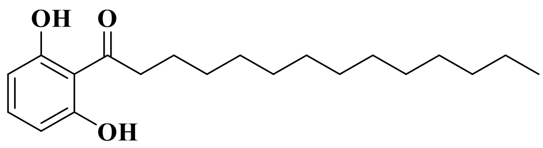


Fraction pool 7-10 on CC separation using Hexane/EtOAc (9.5:0.5, v/v) eluent afforded NS-1 as pale yellow crystalline solid; mp: 91-92 $^{o}$C; FT-IR (neat, $\nu_{max}$, cm$^{-1}$): 3450, 2927, 2852, 1624, 1601, 1448, 1243, 1028, 963, 782, 720; $^{1}$H NMR (500MHz, $CD_3COCD_3$): $\delta$ 11.30 (s, 2H, 2-OH), 7.12 (t, *J*=8Hz, 1H), 6.29 (d, *J*=8Hz, 2H), 3.03 (t, *J*=7.5Hz, 2H), 1.59-1.53 (m, 2H), 1.16 (s, 20H), 0.74 (t, *J*=6.5Hz, 3H, $CH_3$) ppm; $^{13}$C NMR (125MHz, $CD_3COCD_3$); $\delta$ 208.0 (C=O), 162.2, 135.8, 110.1, 107.5, 44.4, 31.7, 24.3, 22.4, 13.4; HR-ESIMS *m/z* 321.24256 $[M+H]^+$ (calcd for $C_{20}H_{32}O_3$, 320.2351).

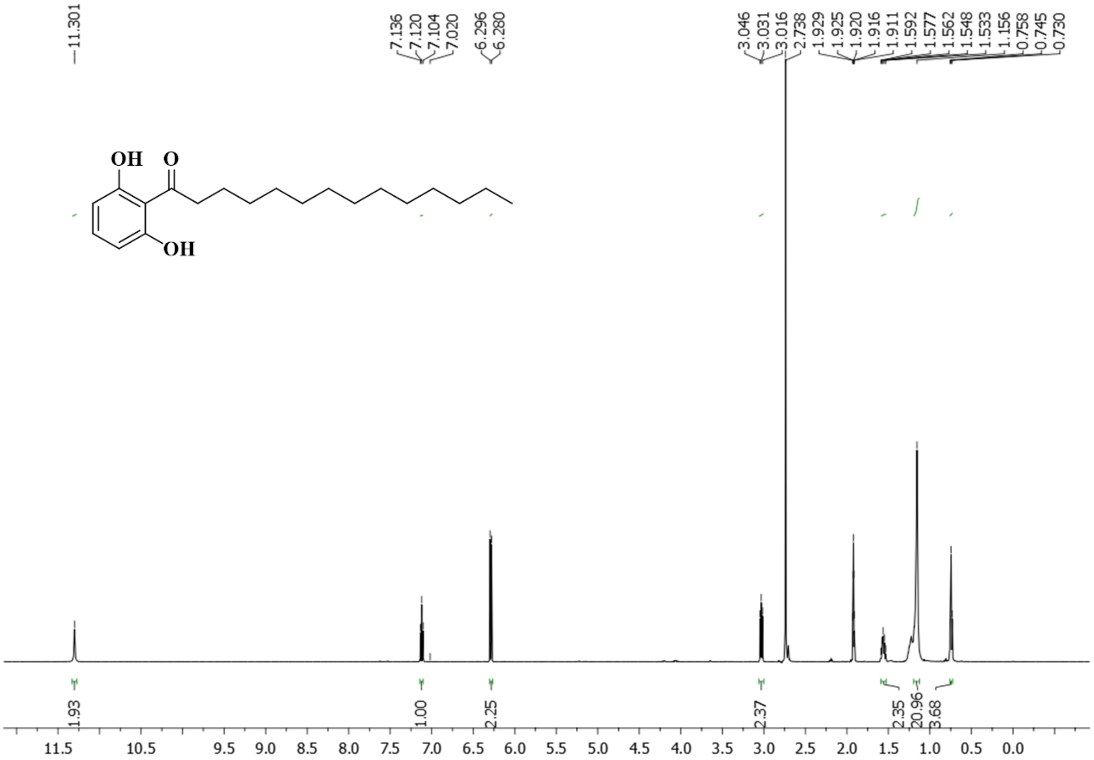


**Figure S2:** $^{1}H$ NMR (500MHz, $CD_3COCD_3$) spectrum of NS-1

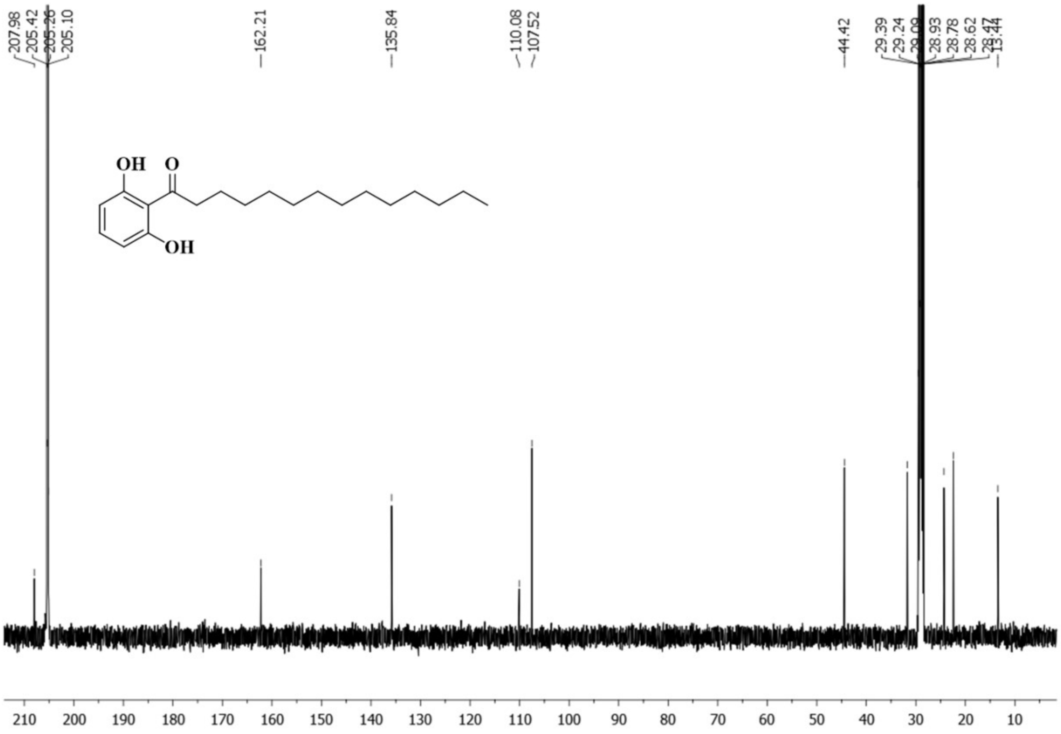


**Figure S3:** $^{13}C$ (125MHz, $CD_3COCD_3$) NMR spectrum of NS-1

Malabaricone A (**NS-3**)

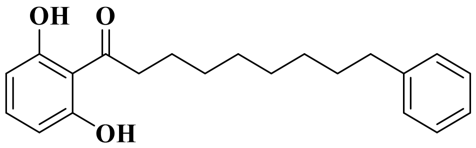


NS-3 was obtained from the fraction pool 11-21 after silica gel column chromatography (CC) using Hexane/EtOAc (9:1, v/v) eluent as yellow crystals; mp: 80-82 $^{o}$C; FT-IR (neat, $\nu_{max}$, $cm^{-1}$): 3254 (-OH), 2925, 2850, 1635 (C=O), 1600, 1471, 1246, 1038, 966, 876.; $^{1}$H NMR (500MHz, $CD_3COCD_3$): $\delta$ 11.44 (s, 2H, 2-OH), 7.28 (dd, $J_1$=2Hz, $J_2$=8Hz, 2H), 7.26 (t, $J$=3Hz, 1H), 7.21 (d, $J$=8Hz, 2H), 7.16 (dd, $J_1$=1.5Hz, $J_2$=7Hz, 1H), 6.43(d, $J$=8.5Hz, 2H, H), 3.17 (t, $J$=7Hz, 2H), 2.62 (t, $J$=7Hz, 2H), 1.74-1.67 (m, 2H), 1.66-1.62 (m, 2H), 1.37 (s, 8H, 4 $CH_2$) ppm; $^{13}$C NMR (125MHz, $CD_3COCD_3$); $\delta$ 208.0 (C=O), 162.2, 142.7, 135.9, 128.3, 128.1, 125.5, 110.2, 107.5, 44.4, 35.6, 31.4, 24.3 ppm; HR-ESIMS *m/z* 349.17833 $[M+Na]^+$ (calcd for $C_{21}H_{26}O_3Na$, 349.1882).

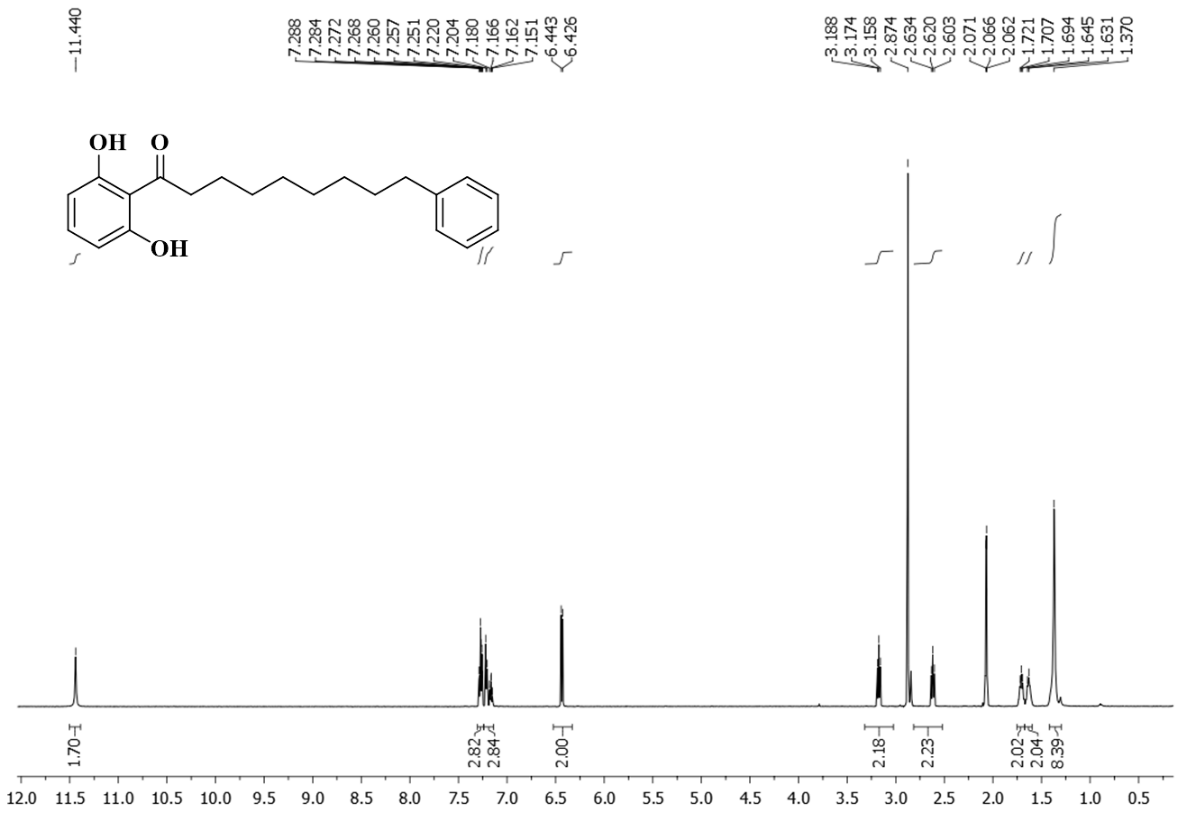


**Figure S4:** $^{1}$H NMR (500 MHz, $CD_3COCD_3$) spectrum of NS-3

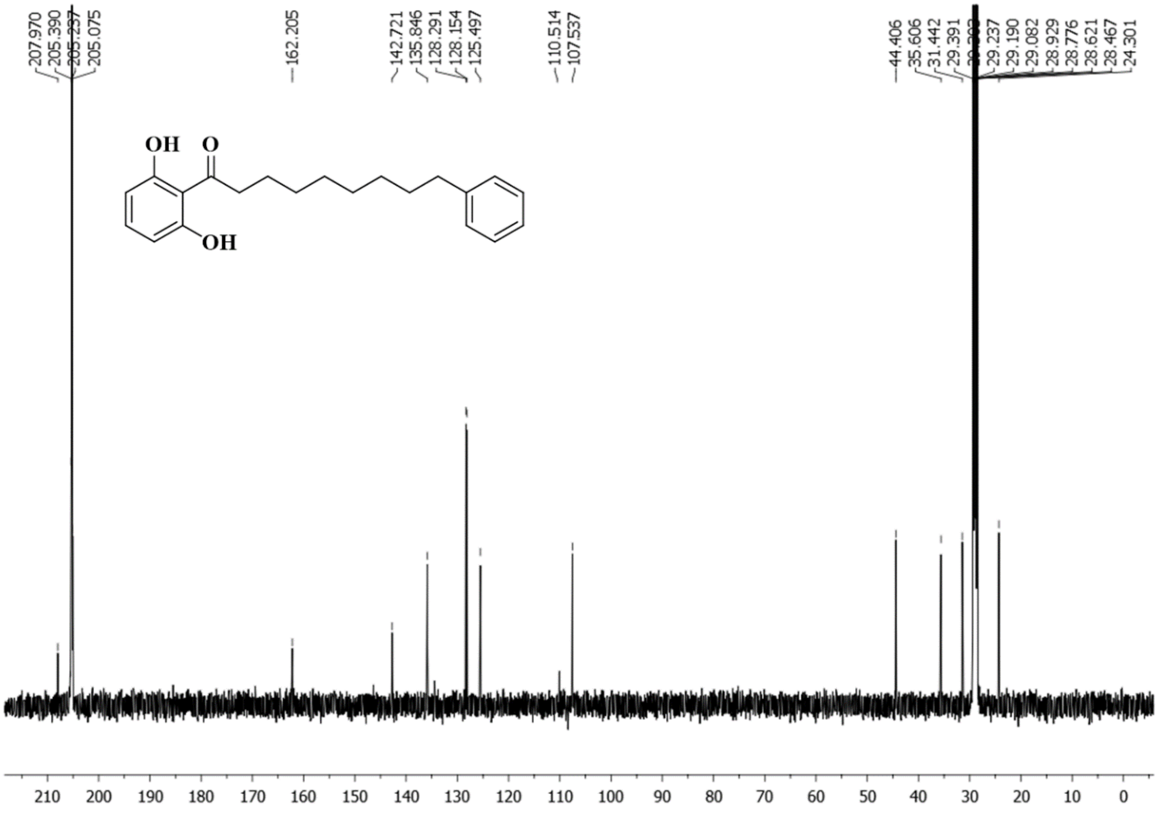


**Figure S5:** $^{13}$C (125MHz, $CD_3COCD_3$) NMR spectrum of NS-3

Promalabaricone B (**NS-5**)

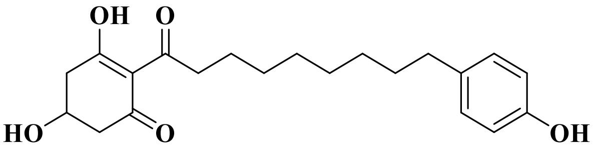


Fraction pool 33-39 subjected to CC separation on silica gel using Hexane/EtOAc (7.5:2.5, v/v) afforded NS-5 as pale yellow crystalline solid; mp: 100-102 °C; FT-IR (neat, $\nu_{max}$, cm$^{-1}$): 3743, 3243, 2912, 2849, 1638, 1542, 1512, 1441, 1363, 1237, 1071, 847, 819, 757,719; $^{1}$H NMR (500MHz, $CD_3COCD_3$): $\delta$ 18.12 (s, 1H, -OH), 7.94 (s, 1H, -OH), 6.88 (d, *J*=7.5 Hz, 2H), 6.60 (d, *J*=7.5Hz, 2H), 4.30 (brs, 1H,-OH), 4.23 (s, 1H), 2.85 (t, *J*=7.5Hz, 2H), 2.71 (s, 2H), 2.60 (d, *J*=17Hz, 2H), 2.38 (t, *J*=8Hz, 2H) 1.45 (m, 4H), 1.20(s, 8H, 4 $CH_2$) ppm; $^{13}$C NMR (125MHz, $CD_3COCD_3$); $\delta$ 197.3, 193.1, 155.3, 133.3, 129.1, 114.9, 112.8, 62.8, 46.8, 41.3, 39.7, 34.7, 31.7, 24.6 ppm; HR-ESIMS *m*/*z* 383.18399 [M+Na]$^{+}$ (calcd for $C_{21}H_{28}O_5Na$, 383.1937).

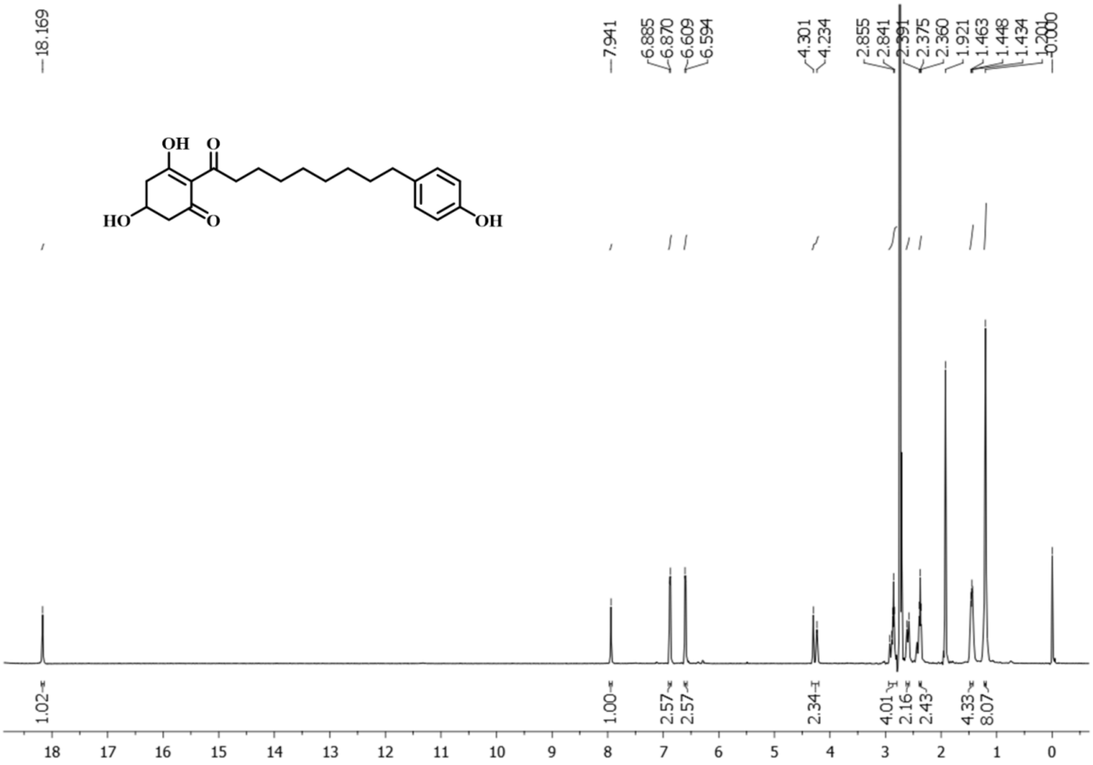

**Figure S6:** $^1$H NMR (500 MHz, $CD_3COCD_3$) spectrum of NS-5

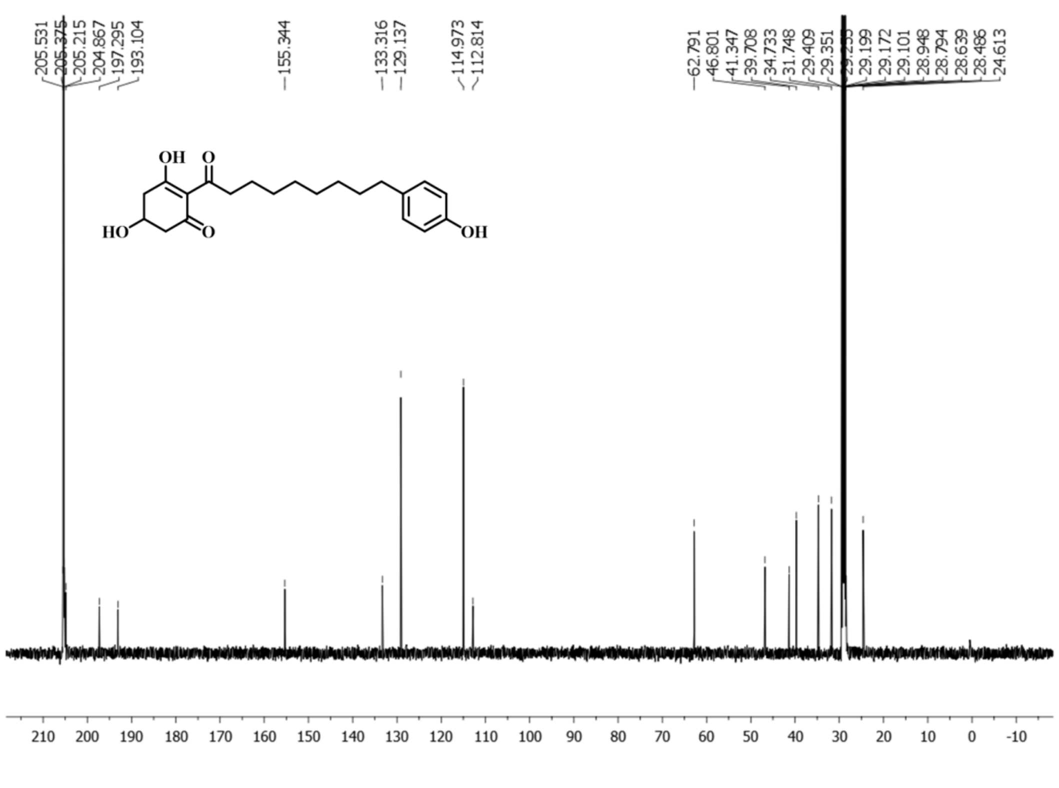

**Figure S7:** $^{13}$C NMR (125 MHz, $CD_3COCD_3$) spectrum of NS-5

Malabaricone B (**NS-7**)

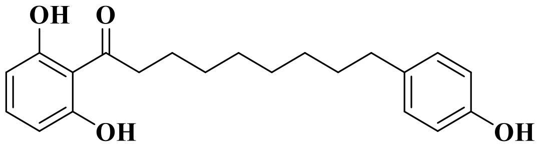


NS-7 was afforded from the fraction pool 22-32 after silica gel column chromatography (CC) using Hexane/EtOAc (7.5:2.5, v/v) eluent as pale yellow solid; mp: 110-112$^{o}$C; FT-IR (neat, $\nu_{max}$, cm$^{-1}$): 3260 (OH), 2920, 2850, 1635 (C=O), 1600, 1471, 1246, 1038, 966, 876; $^{1}$H NMR (500MHz, $CD_3COCD_3$): $\delta$ 11.37 (s, 2H, 2-OH), 8.00 (s, 1H), 7.11 (t, *J*=8Hz, 1H), 6.87 (d, *J*=8.5Hz, 2H), 6.60 (d, *J*=8.5Hz, 2H), 6.29 (d, *J*=8.5Hz, 2H), 3.02 (t, *J*=7Hz, 2H), 2.37(t, *J*=7.5Hz, 2H), 1.56-1.53 (m, 2H), 1.46-1.40 (m, 2H, H-8)1.21 (s, 8H, 4 $CH_2$) ppm; $^{13}$C NMR (125MHz, $CD_3COCD_3$); $\delta$ 208.0 (C=O), 162.2, 155.4, 135.8, 133.3, 129.1, 115.0, 110.1, 107.5, 44.4, 34.7, 31.7, 24.3 ppm; HR-ESIMS *m*/*z* 365.17291 [M+Na]$^{+}$ (calcd for $C_{21}H_{26}O_4Na$, 365.1831).

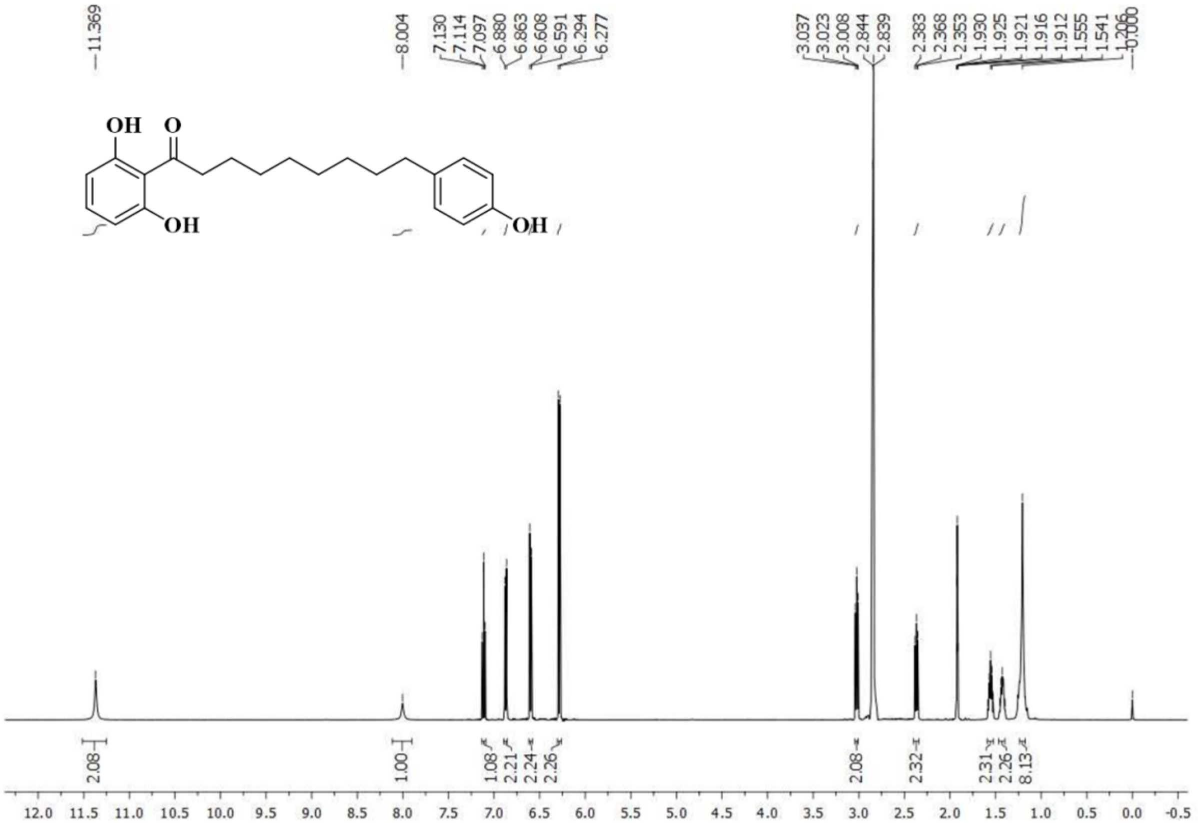


**Figure S8:** $^{1}$H NMR (500 MHz, $CD_3COCD_3$) spectrum of NS-7

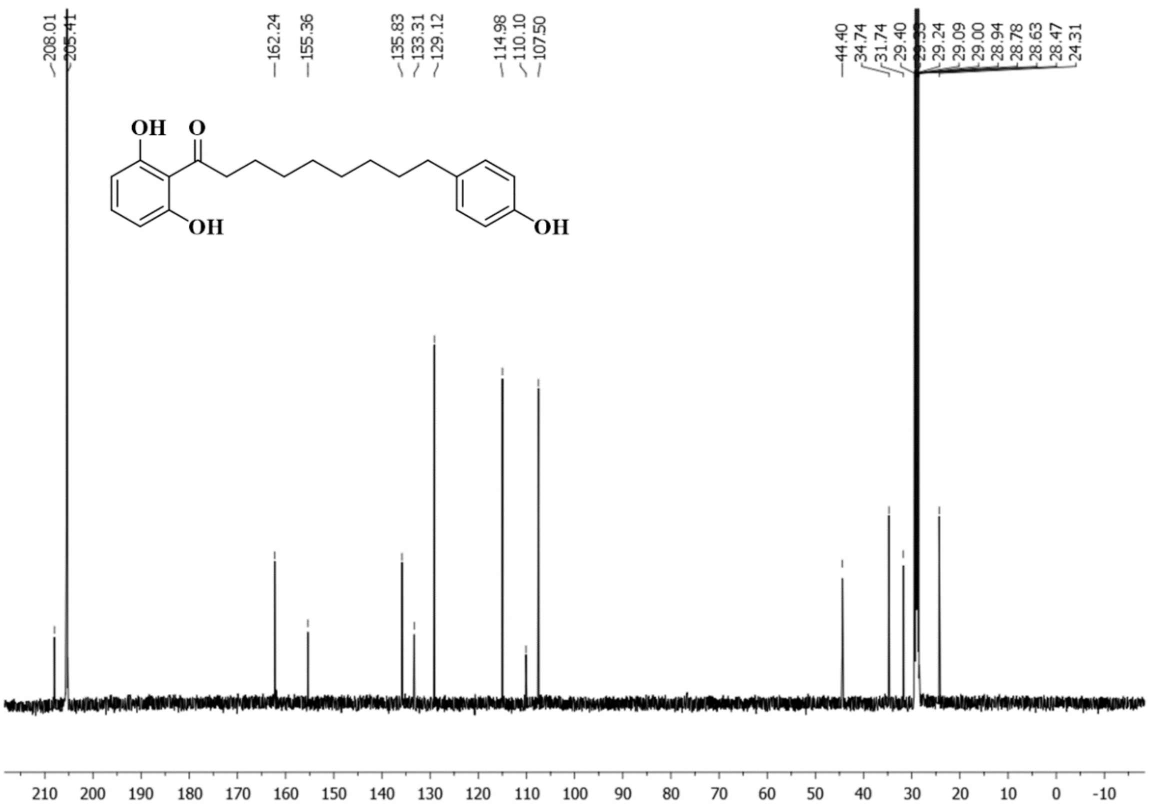


**Figure S9:** $^{13}C$ NMR spectrum (125 MHz, $CD_3COCD_3$) of NS-7

Malabaricone C (**NS-9**)

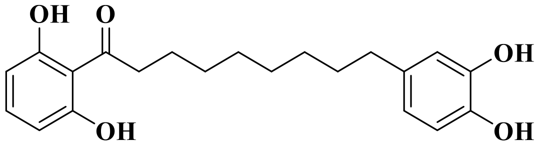


Fraction pool 40–54 was purified by column chromatography by eluting Hexane/ EtOAc (6.5:3.5, v/v) as eluent followed by crystallization using DCM/hexane afforded NS-9 as yellow crystalline solid; mp: 122-124 $^{o}$C; FT-IR (neat, $\nu_{max}$, $cm^{-1}$): 3347, 2962, 2849, 2349, 1865, 1623, 1592, 1517, 1432, 1340, 1246, 1120; $^{1}H$ NMR (500MHz, $CD_3COCD_3$): $\delta$ 11.45 (s, 2H, -OH), 7.63 (s, 2H, -OH), 7.24 (t, $J$=8Hz, 1H), 6.70 (d, $J$=8Hz, 1H), 6.67 (d, $J$=2.5Hz, 1H), 6.50 (dd, $J_1$=2Hz, $J_2$=8Hz, 1H), 6.42 (d, $J$=8Hz, 2H), 3.15 (t, $J$=8Hz, 2H), 2.44 (t, $J$=7.5Hz, 2H), 1.67-1.55 (m, 2H), 1.54-1.29 (m, 2H), 1.28 (s, 8H, 4 $CH_2$) ppm; $^{13}C$ NMR (125MHz, $CD_3COCD_3$); $\delta$ 208.1 (C=O), 162.3, 144.9, 142.9, 135.9, 134.3, 119.4, 115.3, 115.0, 110.1, 107.4, 34.9, 31.7, 24.3 ppm; HR-ESIMS $m/z$ 381.16666 $[M+Na]^+$ (calcd for $C_{21}H_{26}O_5Na$, 381.1780).

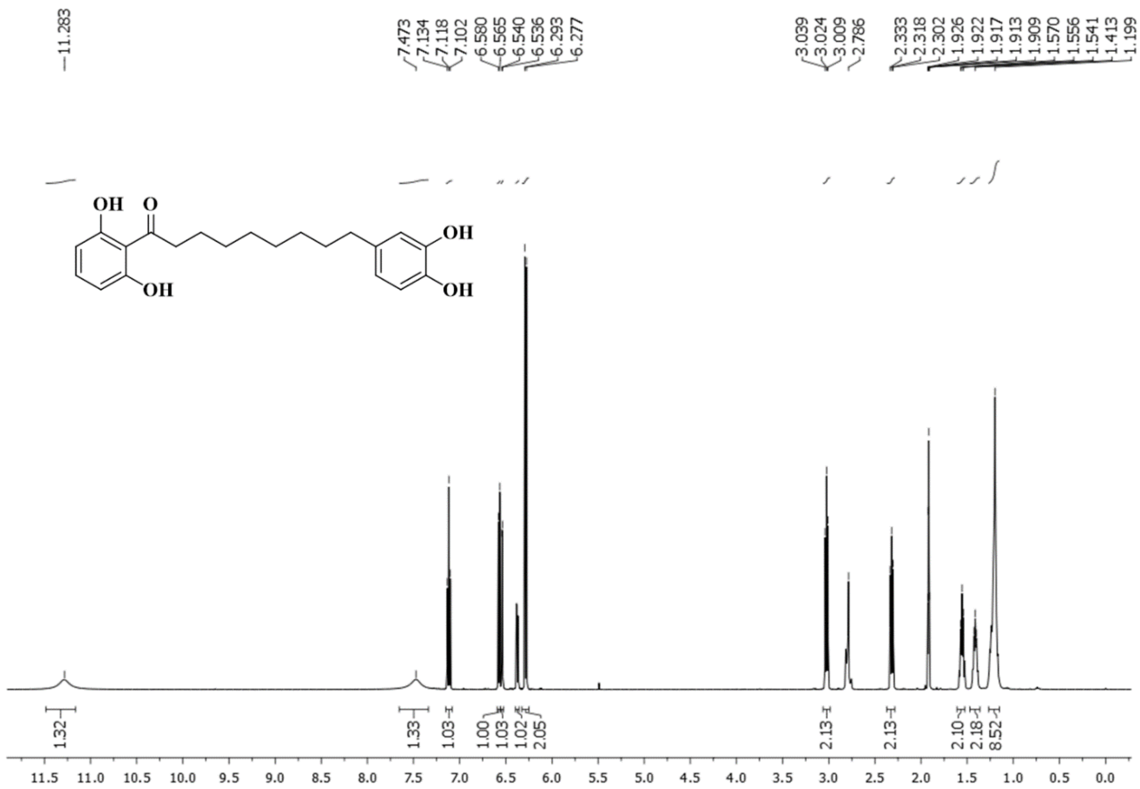

**Figure S10:** $^{1}$H NMR (500MHz, $CD_3COCD_3$) spectrum of NS-9

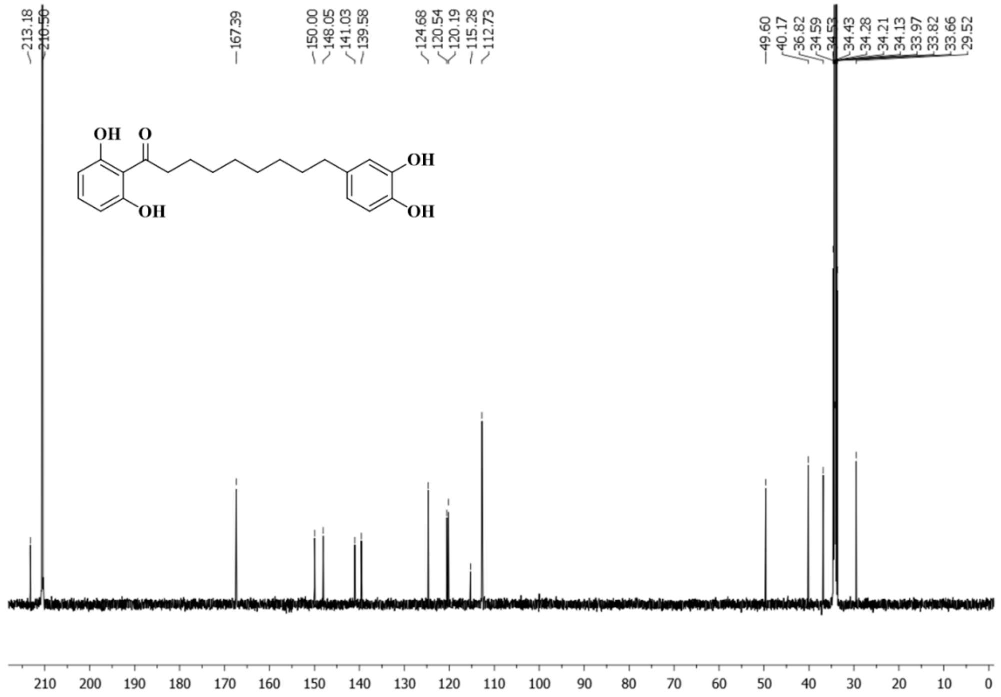

**Figure S11:** $^{13}$C NMR (125 MHz, $CD_3COCD_3$) spectrum of NS-9

Malabaricone D (**NS-11**)

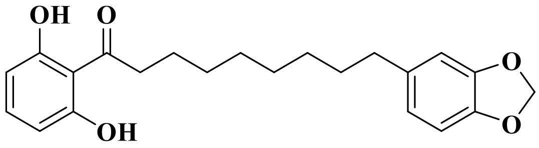


NS-11 was also isolated as pale yellow crystalline solid from the CC separation of the fraction pool 11-21 using gradient elution of Hexane/EtOAc (9.5:0.5 to 9:1, v/v); mp: 68-70 $^{o}$C; FT-IR (NaCl, $\nu_{max}$, cm$^{-1}$): 2924, 2843, 1622 (C=O), 1588, 1492, 1449, 1380, 1362, 1329, 1293, 1249, 1176, 1124, 1091, 1037, 961,938,917, 872, 840, 790, 749, 717, 657; $^{1}$H NMR (500MHz, $CD_3COCD_3$): $\delta$ 11.33 (s, 2H, -OH), 7.12 (t, *J*=8.5Hz, 1H), 6.59 (d, *J*=8 Hz, 1H), 6.52 (d, *J*=7.5Hz, 1H), 6.29 (d, *J*=8.5Hz, 2H), 5.79 (s, 2H, -O-$CH_2$-O-), 3.02 (t, *J*=7.5Hz, 2H), 2.40 (t, *J*=8Hz, 2H), 1.58-1.52 (m, 2H), 1.45-1.41 (m, 2H), 1.21 (s, 8H, 4 $CH_2$) ppm; $^{13}$C NMR (125MHz, $CD_3COCD_3$); $\delta$ 207.9 (C=O), 162.3, 147.6, 145.5, 136.6, 135.9, 121.0, 110.1, 108.6, 107.6, 107.5, 100.7(-O-$CH_2$-O), 44.4, 35.3, 31.7, 29.4, 29.3, 24.3 ppm; HR-ESIMS *m/z* 393.16763 [M+Na]$^{+}$ (calcd for $C_{22}H_{26}O_5Na$, 393.1678).

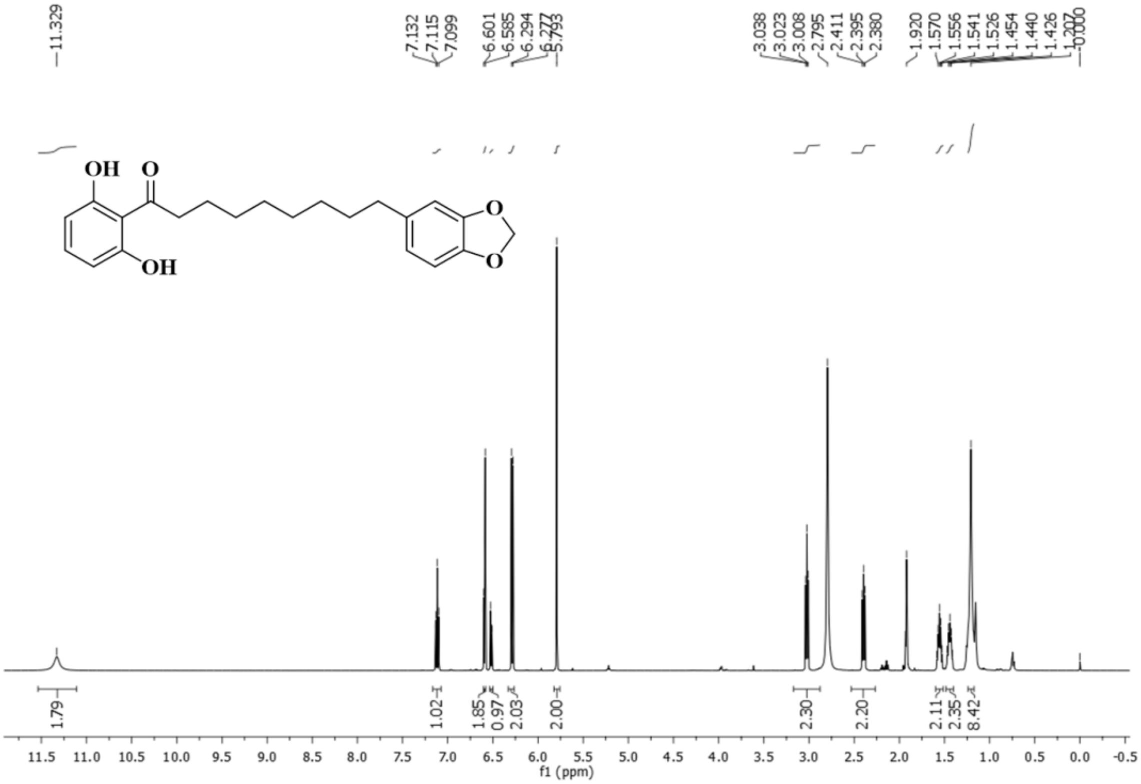


**Figure S12:** $^{1}$H NMR (500MHz, $CD_3COCD_3$) spectrum of NS-11

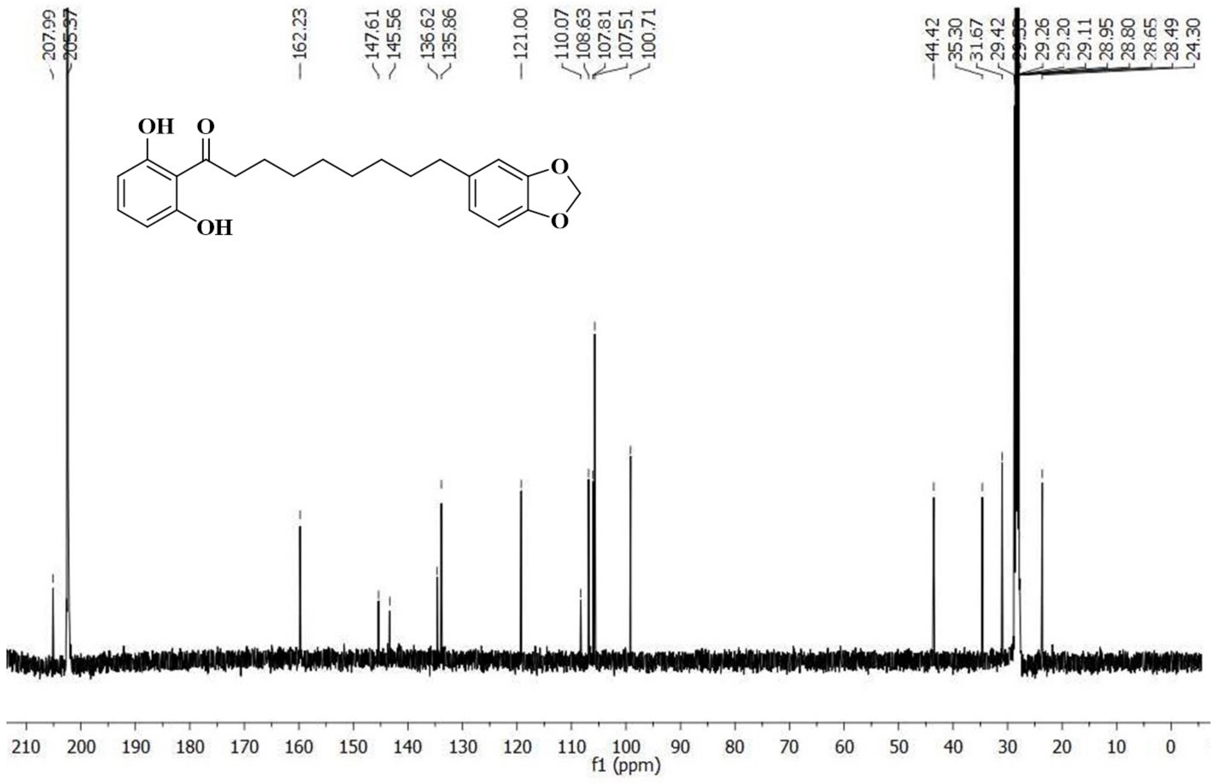


**Figure S13:** $^{13}C$ (500MHz, $CD_3COCD_3$) NMR spectrum of NS-11
